# An Inflammation Loop Orchestrated by S100A9 and Calprotectin Is Critical for Development of Arthritis

**DOI:** 10.1371/journal.pone.0045478

**Published:** 2012-09-18

**Authors:** Annabelle Cesaro, Nadia Anceriz, Audrey Plante, Nathalie Pagé, Mélanie R. Tardif, Philippe A. Tessier

**Affiliations:** Centre de recherche en Infectiologie, Centre de recherche du Centre Hospitalier Universitaire de Quebec, and Faculty of Medecine, Université Laval, Quebec, Canada; Institut Jacques Monod, France

## Abstract

**Objective:**

The S100A9 and S100A8 proteins are highly expressed by neutrophils and monocytes and are part of a group of damage-associated molecular pattern molecules that trigger inflammatory responses. Sera and synovial fluids of patients with rheumatoid arthritis (RA) contain high concentrations of S100A8/A9 that correlate with disease activity.

**Methods:**

In this study, we investigated the importance of S100A9 in RA by using neutralizing antibodies in a murine lipopolysaccharide-synchronized collagen-induced arthritis model. We also used an *in vitro* model of stimulation of human immune cells to decipher the role played by S100A9 in leukocyte migration and pro-inflammatory cytokine secretion.

**Results:**

Treatment with anti-S100A9 antibodies improved the clinical score by 50%, diminished immune cell infiltration, reduced inflammatory cytokines, both in serum and in the joints, and preserved bone/collagen integrity. Stimulation of neutrophils with S100A9 protein led to the enhancement of neutrophil transendothelial migration. S100A9 protein also induced the secretion by monocytes of proinflammatory cytokines like TNFα, IL-1β and IL-6, and of chemokines like MIP-1α and MCP-1.

**Conclusion:**

The effects of anti-S100A9 treatment are likely direct consequences of inhibiting the S100A9-mediated promotion of neutrophil transmigration and secretion of pro-inflammatory cytokines from monocytes. Collectively, our results show that treatment with anti-S100A9 may inhibit amplification of the immune response and help preserve tissue integrity. Therefore, S100A9 is a promising potential therapeutic target for inflammatory diseases like rheumatoid arthritis for which alternative therapeutic strategies are needed.

## Introduction

Rheumatoid arthritis (RA) is a chronic systemic inflammatory disease characterized by a massive infiltration of immune cells into the synovial lining, initiating local inflammation and ultimately leading to cartilage/bone destruction. Although the causes of RA remain unknown, multiple pro-inflammatory mediators actively participate in the progression and severity of the disease. These molecules include cytokines and chemokines like tumor necrosis factor-alpha (TNFα), interleukin (IL)-6, and macrophage inflammatory protein (MIP)-1α [1], [2], which trigger a feed-forward loop sustaining inflammation in synovial joints. Inhibitors of some of these cytokines, like TNFα or IL-6, are routinely used clinically to treat RA. While these treatments are effective, various side effects have been reported [3], [4], [5]. Moreover, approximately 30% of patients do not respond efficiently to the treatment, while others become resistant over time [6].

S100A9 and S100A8 are small calcium-binding proteins recognized as damage-associated molecular pattern (DAMP) molecules [7] upon their release in the extracellular environment. They are increased in the serum and upregulated the synovium of RA patients and their levels correlate with disease severity [7], [8], [9], [10]. This increase is observed not only in RA but also in other inflammatory diseases like inflammatory bowel disease [11], [12] and gout [13], [14]. S100A8 and S100A9 are primarily expressed in innate immune cells, particularly in neutrophils, constituting approximately 40% of the cytosolic proteins in these cells. They are also expressed, albeit to a lesser extent, in monocytes [15], [16]. They can form homo- or hetero-complexes, the latter known as calprotectin. Both forms are abundantly released by neutrophils and monocytes under stress or inflammatory conditions [17], [18]. Signaling pathways that are induced upon sensing these molecules trigger inflammatory responses such as chemotaxis [14], [19], phagocyte migration [20], and modulation of various macrophage functions. Based on their involvement in inflammatory processes and abundant levels in numerous pathologies, it is likely that S100A9 and calprotectin play a pivotal role in the pathophysiology of various inflammatory disorders. In this study, we used a combination of *in vivo* and *in vitro* experiments to gain new insights into the proinflammatory activities of S100A9 and investigate the impact of anti-S100A9 therapy on acute arthritis development.

## Materials and Methods

### Protein and Antibody Production

**Figure 1:**
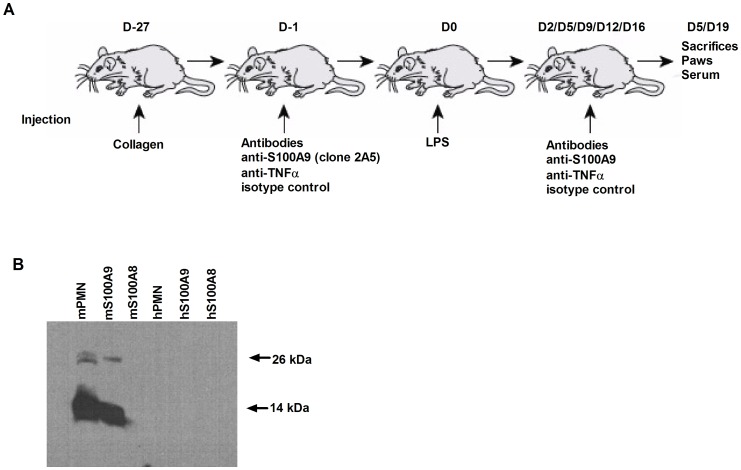
LPS-CIA model. (A) Mice (25 mice/group) were immunized at the base of the tail with 100 µg of chicken type II collagen on day “−27” and then injected i.p. with LPS (25 µg in PBS) on day 0. Two milligrams of neutralizing Abs or isotype control Abs were injected into mice 24 h before LPS injection. During the protocol, five injections were given, at 2, 5, 8, 12, and 16 days post-LPS injection. On day 5 and 19, mice were sacrificed to collect the paws and the serum for further analysis. (B) Murine monoclonal anti-S100A9 (Clone 2A5) was tested for specificity by western blot against murine protein mS100A9, mS100A8, a lysate of murine neutrophils, human protein hS100A12, hS100A9, hS100A8 and a lysate of human neutrophils.

Murine S100A9 monoclonal antibody (mAb) was generated using the ClonaCell® – H hybridoma cloning kit (Stemcell Technologies) according to the manufacturer’s instructions. Briefly, Sprague Dawley rats (Charles River) were immunized four times with full length murine S100A9 protein. Spleen and myeloma cells were mixed and plated on a methylcellulose-based selection medium. Individual colonies were picked using a pipette, transferred into a 96-well plate and tested for mAb secretion. Clone 2A5 (IgG1κ) was selected based on its ability to inhibit recruitment in vivo in response to murine S100A9. Clone 2A5 was cultured in CeLLine™ flasks (BD Biosciences) for Ab production. The supernatant was collected after 14 days and purified using the Pierce® Thiophilic Adsorbent method. Purified rabbit IgGs directed against murine S100A9 and S100A8 and recombinant human S100A9 (rhS100A9) were prepared as previously described [14]. The absence of endotoxin contamination in Ab and protein preparations was confirmed using the Limulus amebocyte assay (Cambrex) for Abs and proteins before use. Antibody concentration for clone 2A5 was determined by Bradford protein assay (Bio-rad). Specificity of the murine monoclonal antibody was confirmed by western blot (Fig. 1 B). Clone 2A5 used in all in vivo experiments recognized only murine S100A9.

### Induction of LPS-CIA

All experiments were carried out in accordance with the Laval University animal protection committee (Sainte-Foy, Quebec, Canada). Six- to 8-week-old female DBA/1 mice, obtained from Jackson Laboratories were immunized subcutaneously at the base of the tail with chicken collagen type II (100 µg/mouse, Chondrex inc) emulsified in complete Freund’s adjuvant, prior to receiving an i.p. injection of LPS (25 µg/mouse) 27 days later according to the manufacturer’s instructions (Fig. 1A). Disease activity was monitored every other day on a scale of 0 to 4 per paw (0, normal; 1, mild, but definite redness and swelling of the ankle or wrist, or apparent redness and swelling limited to individual digits, regardless of the number of affected digits; 2, moderate redness and swelling of ankle of wrist; 3, severe redness and swelling of the entire paw including digits; 4, maximally inflamed limb with involvement of multiple joints) for a maximum disease score of 16 per mouse. To evaluate the effects of S100A9-neutralizing Abs on LPS-CIA development, mice were injected with 2 mg of Abs 24 h before LPS injection, and then injected twice a week with 1 mg of Abs until the end of the protocol. An anti- TNFα (clone MP6-XT3, Upstate Cell Signaling) and a rat isotype control treatment (ChromPure Rat IgG, whole molecule, Jackson Immuno Research) antibody were used as positive and negative controls, respectively (Fig. 1A).

### Histopathological Assessment of LPS-CIA

For histopathology, the paws were removed and fixed in paraformaldehyde 4%, decalcified in TBD-2 Thermo-Shandon solution (Thermo Fisher Scientific) for 10 days, and embedded in paraffin. Sections were stained with H&E (Thermo Fisher Scientific) or safranin/fast green colorations (VWR International). Bone destruction, collagen integrity, and cell infiltration were evaluated by two blinded observers according to the following scales: 0–3 for bone destruction (0, normal; 1, slight degradation; 2, mild degradation; 3, complete destruction); 0–2 for collagen (0, normal; 1, mild loss; 2, complete loss); 0–2 for cell infiltration (0, normal; 1, mild infiltration; 2, severe infiltration).

### Immunohistochemistry

Paws were cut into sections 5 µm thick. The deparaffinized and rehydrated sections were incubated with hydrochloric acid for antigen retrieval. After blocking endogenous peroxidase activity and non-specific binding using the ready to use Protein Block Serum free (Dako) slides were incubated with rabbit anti-S100A9, rabbit anti-S100A8 (10 µg/ml) or preimmune serum (1/1000) diluted in Tris-buffered saline overnight at 4°C. After washings, tissue sections were incubated with anti-rabbit IgG HRP diluted in Tris-buffered saline for 1 hour at RT. Detection was performed using the ready-to-use AEC solution (Dako). The slides were then counterstained with Mayer’s haematoxylin according to the manufacturer’s instructions (Dako).

### Western Blot Analysis

Paws of sacrificed mice (5 days, at the top of inflammation) were cut at the ankle or wrist and frozen in dry ice. Fore and hind paws were separately homogenized using a polytron homogenizer in 1ml of RIPA buffer with a protease inhibitor cocktail. The homogenates were centrifuged at 1200 rpm for 5 min, and supernatants were then further centrifuged at 13,000 rpm for 15 min. Thirty microliters from a pool of 5 supernatants mixed in sample buffer were loaded on SDS-PAGE (15%). Proteins were detected using anti-7/4 Ab (R&D Systems), anti-Gr1 Ab (BD Biosciences), anti-IL-6 Ab (R&D Systems), or anti-TNFα Ab (PeproTech).

### ELISAs

Murine blood was collected at 4 h, and 5 days after LPS injection, and centrifuged to obtain serum. Serum anti-collagen II antibody titers were measured using an anti-collagen detection kit according to the manufacturer’s instructions (Chondrex inc). S100A9 and calprotectin were quantified by a homemade ELISA kit. Briefly, plates were coated with 100 µl anti-S100A9 mAb (clone 2A5, 2 µg/ml) and left overnight at 4°C. After incubation, plates were washed three times and blocked with PBS/0.1% Tween-20/2% BSA for 1 h at room temperature (RT). Samples and standards were added and incubated for 1 h at RT. The plates were then washed and incubated with S100A8 polyclonal Ab (5 µg/ml) for 1 h at RT, for calprotectin detection, respectively. Following incubation, plates were washed and incubated with anti-rabbit IgG at a dilution of 1∶5000 and revealed by using TMBS (Neogen Corporation), according to the manufacturer’s instructions. ELISA kits for murine IL-6 were obtained from PeproTech and used according to the manufacturer’s instructions within the range of 62–4000 pg/ml.

### Transmigration Assay

Transwell chambers with a 6.5 mm diameter polycarbonate filter with a pore size of 3 µm (Corning) were used. HUVECs (ATCC) were grown to confluence on filters coated with 0.5% gelatine in PBS (Sigma-Aldrich). Confluence was confirmed before each experiment by measuring resistance using an ohmmeter. Neutrophil were obtained from heathly donnors and purified by centrifugation on Ficoll-Paque cushions (Wisent, inc) [19]. Fifty microliters of S100A9 or buffer (M199-5mM HEPES) and 50 µl of calcein-AM-labeled neutrophils were added to the upper well. IL-8 (5 ng/ml in M199-5 mM HEPES) or buffer was added to the lower chamber and cells were allowed to migrate for 2 h at 37°C. After this incubation period, wells were gently shaken to dislodge the last transmigrated cells and the cells in the lower chambers were lysed by adding 150 µl Triton X-100 (5%) for 20 min in the dark and fluorescence was measured.

### Adhesion to Fibrinogen

Immunoassay plates (96-well, Corning Inc., Corning, NY, USA) were coated overnight at 4°C with 50 µl of fibrinogen (2 mg/ml) in NaHCO_3_ 0.1 M, pH 9.6. The plates were washed three times with 100 µl of HBSS-H (HBSS supplemented with 10 mM HEPES, pH 7.4) containing 1.3 mM Ca^2+^ and 0.8 mM Mg^2+^) before use. Fifty microliters of each stimulus solution (S100A9 at 80 µg/ml = 2×, IL8 at 10 ng/ml = 2×, alone or in combination) diluted in HBSS-H, were added to individual wells. Neutrophils were resuspended at 5 × 10^6^ cells/ml in HBSS-H and labelled with 5 µM of the intracellular fluorescent dye calcein-AM (Calbiochem) for 20 min at 37°C. After washing, 50 µl of the cell suspension (5 × 10^6^ cells/ml) was added to each well. Plates were centrifuged for 2 min at 250 ×g and neutrophils were allowed to adhere for increasing periods of time at 37°C before being washed three times by immersion in cold PBS. Adherent cells were lysed by adding deionised distilled water, and fluorescence was measured at λ_ex_ = 485 nm and λ_em_ = 530 nm using a fluorescence plate reader.

### Blocking Experiments

Transmigration assays were performed as described in the material and methods section. Anti-S100A9, anti-CD11a, anti-CD11b, anti-CD18, or anti-ICAM-1 were mixed with neutrophils in the upper well during migration assays.

### Stimulation of Monocytes with S100A9 Proteins

Monocytes from healthy donors were purified using the Human Enrichment Cocktail for RosetteSep® kit (StemCell Technologies) according to the manufacturer’s instructions. Cell suspensions were adjusted to a density of 1 × 10^6^ cells per ml in RPMI containing 10% FBS and incubated in 48-well plates (Corning Life Sciences) with S100A9 (0.1–40 µg/ml). Culture supernatants were harvested 24 h later and tested for cytokine secretion. For some experiments, a time course (0–24 h) was also carried out. Cytokine arrays (R&D Systems) and ELISA for IL-6, TNFα, or MIP-1α (PeproTech) were performed according to the manufacturer’s instructions.

### Statistical Analyses

Anti-collagen, S100A9, S100A8/A9 concentrations, collagen/bone destruction, and cell infiltration were compared using one-way ANOVA followed by Tukey’s multiple comparison test of the means. A p-value <0.05 was considered statistically significant. Secretion of murine or human cytokines was compared by t test. Protective efficacies of the different treatments were evaluated by comparing arthritis clinical scores using repeated measures ANOVA. Neutrophil migration was compared using one-way ANOVA followed by Dunett’s multiple comparison test. A p-value <0.05 was considered statistically significant.

## Results

### S100A9 and Calprotectin are Rapidly Secreted during Arthritis Development

**Figure 2:**
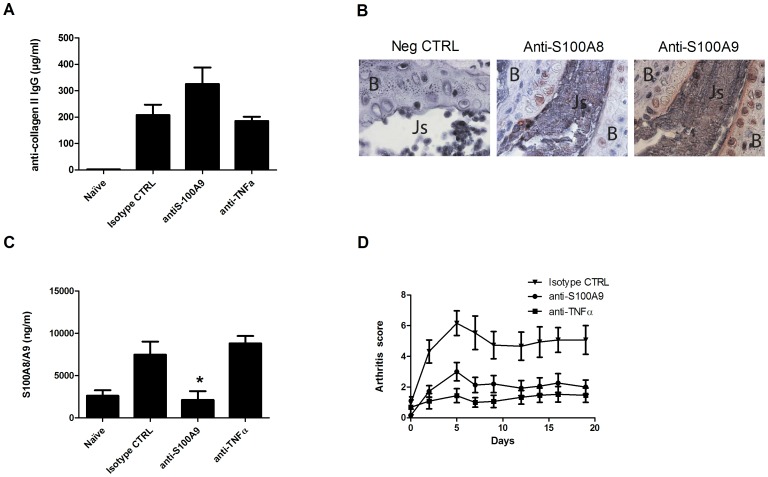
Effect of anti-S100A9, TNFα, or isotype control Abs on the LPS-CIA clinical score. (A) Anti-collagen II Abs were measured in mouse serum 4 h after LPS injection. The results are expressed as µg of IgG Ab/ml of serum. (B) S100A8 and S100A9 expression in arthritic paws. Paw tissue sections were stained with rabbit pre-immune serum, rabbit anti-S100A8, or rabbit anti-S100A9 polyclonal IgGs. B: bone, Js: joint space. (Magnification 1000X). (C) Four hours after LPS injection, sera were collected and ELISA S100A8/A9 were performed *p<0.05, one-way ANOVA test (n = 10 paws/group), Tukey’s Multiple Comparison Test (D) Clinical score as assessed by two blinded observers. Data are the mean scores calculated from at least 15 mice per group until day 19.

Collagen-induced arthritis (CIA) is commonly used as a model of RA because it shares immunological and pathological features with human RA [21], [22]. Here we chose the murine lipopolysaccharide (LPS)-CIA model to synchronize arthritis within experimental groups, as all animals develop arthritis within 24h following LPS injection. To ensure that all groups synthesize similar levels of anti-collagen II, the sera of 10 mice from each of the different groups were collected 4 h after the LPS boost to perform an enzyme-linked immunosorbent assay (ELISA). As expected, all groups showed similar concentrations of anti-collagen II ranging from 185 to 326 µg immunoglobulin G (IgG)/ml (Fig. 2A, not statistically significant between groups). These results confirmed that arthritis was similarly triggered in all groups, and that further observations may be attributed to specific treatments received by each group.

S100A8 and S100A9 are known to be expressed in the synovium of RA patients [23]. We found expression of both proteins in paw of arthritic mouse in the CIA models (Fig. 2B). Because S100A9 and S100A8/A9 are early markers of inflammation in various chronic inflammatory diseases including RA [7], [8], [9], [10], we used ELISA to quantify calprotectin in the serum of mice collected 4 h after LPS injection. The expression of calprotectin was strongly upregulated in the isotype control-treated and anti-TNFα-treated groups compared to naïve mice (Fig. 2C). As expected, a strong decrease in the levels of S100A8/A9 proteins was observed in mice treated with the anti-S100A9 antibody (2.1±1.0 µg/ml for S100A8/A9, ), compared to levels seen in mice treated with the isotype control Ab (and 7.5±1.5 µg/ml).

### Anti-S100A9 Decreases Arthritis Severity, Bone/cartilage Destruction, and Cell Infiltration

Arthritis severity in the murine LPS-CIA model was scored on a scale of 0 to 4 per paw, with a maximum possible score of 16 per animal, based on inflammation markers (redness and swelling). Mice treated with anti-S100A9 showed markedly decreased arthritis severity scores compared to the isotype control group. The first signs of inflammation such as redness or slight swelling were detected at approximately day 2 and disease activity was maximal on day 5 after LPS boost. Anti-S100A9 and anti-TNFα treatment significantly reduced the maximum arthritis score median, from 6.8 in the isotype control-treated group to 3 and 1.4 in groups treated with anti-S100A9 and anti-TNFα, respectively (Fig. 2D). Overall, anti-S100A9 treatment led to an approximately 50% reduction in disease intensity (Fig. 2D). Disease incidences in groups treated with anti-TNFα, anti-S100A9, and the isotype control were 53%, 60%, and 80%, respectively (data not shown). Statistical analysis revealed significant differences between anti-S100A9 or anti-TNFα-treated and isotype control groups. No significant difference in disease activity was observed between anti-S100A9 and anti-TNFα-treated animals.

**Figure 3:**
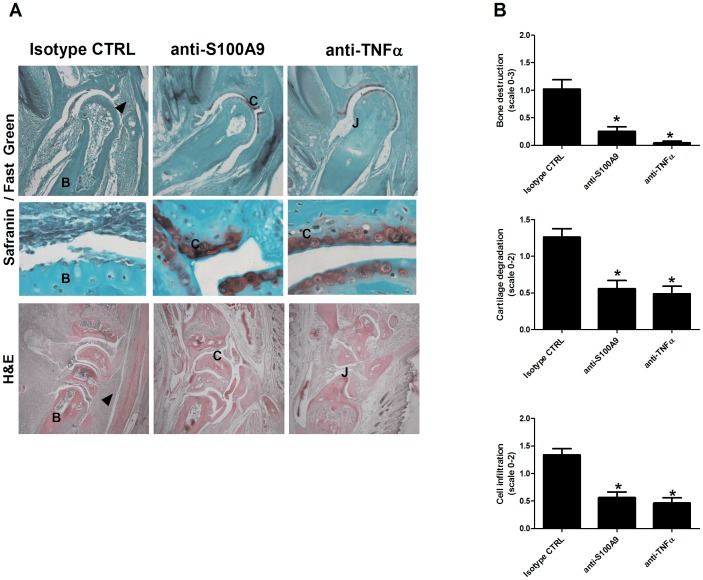
Histological assessment of joint infiltration and destruction in anti-S100A9-, anti-TNFα-, and isotype control-treated animals. (A) H&E-stained sections of wrist at 40× magnification and safranin/fast green-stained sections of the distal phalange joints at 100× magnification. The enlargement was taken at 1000 × magnification. B: bone, C: cartilage, J: joint. (B) Bone destruction, collagen degradation, and cell infiltration as assessed by two blinded observers (on a scale of 0–3, 0–2, 0–2, respectively). *p<0.05, one-way ANOVA test (n  = 10 paws/group, 5 forepaws and 5 hind paws), Tukey’s multiple comparison test.

As in RA, the murine LPS-CIA model is characterized by strong infiltration of leukocytes into joint articulations, as well as by marked degradation of bone and collagen. In light of the improvement in clinical arthritic scores following anti-S100A9 therapy, we examined the effect of anti-S100A9 on cellular recruitment and joint integrity. To this end, paws were recovered at the peak of inflammation on day 5. Paw sections from 5 animals per group were stained with hematoxylin and eosin (H&E) to determine cellular infiltration and joint architecture, as well as with safranin/fast green to assess collagen degradation. Mice treated with the isotype control Ab exhibited marked bone destruction with a loss of collagen demonstrated by a decrease in safranin staining (Fig. 3A). In comparison, mice treated with anti-S100A9 showed almost no collagen loss or bone degradation. Blinded analyses of sections confirmed that anti-S100A9 treatment markedly reduced bone destruction (mean scores of 0.25 vs. 1.02 for anti-S100A9 and isotype control treatments, respectively) as well as collagen destruction (mean scores of 0.55 vs. 1.26 for anti-S100A9 and isotype control treatments, respectively, p>0.05, one-way analysis of variance [ANOVA] test). Similar results were obtained with anti-TNFα-treated mice (Fig. 3B, no significant differences between anti-S100A9 and anti-TNFα-treated animals). These results indicate that treatment with anti-S100A9 protects against joint destruction.

**Figure 4:**
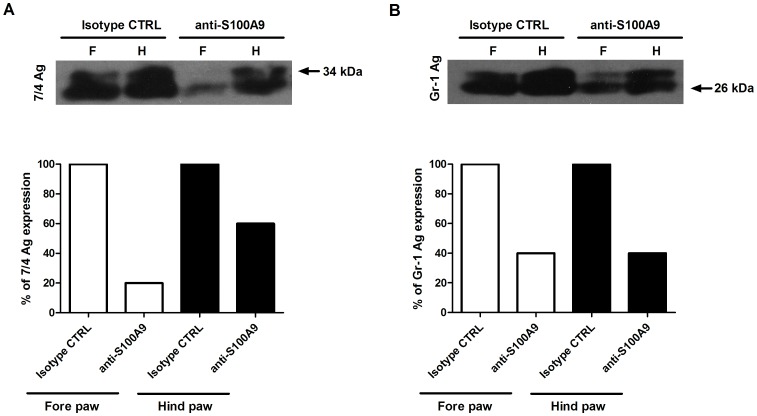
Decreased neutrophil and monocyte antigen expression in the paws of anti-S100A9-treated mice. (A) Western blot analysis of 7/4 antigen (Ag), a neutrophil marker (top). Quantification of 7/4 Ag on an immunoblot by densitometry analysis (bottom). (B) Western blot analysis of Gr-1 Ag, a marker of granulocytes and monocytes (top). Quantification of Gr-1 Ag on an immunoblot by densitometric analysis (bottom).

H&E staining of hind and fore paws revealed a significant decrease in cellular infiltration into the synovium in anti-S100A9-treated animals (mean scores of 0.56 vs. 1.33 for anti-S100A9 and isotype control treatments, respectively; Fig. 3). Western blot analyses of paw homogenates showed a 40% to 80% reduction in the expression of the neutrophil marker 7/4 in anti-S100A9-treated animals, compared to animals treated with the isotype control Ab (Fig. 4A). Similarly, a 60% reduction in the monocyte/granulocyte marker Gr-1 was observed in anti-S100A9-treated animals (Fig. 4B).

### Anti-S100A9 Prevents the Release of Pro-inflammatory Cytokines

**Figure 5:**
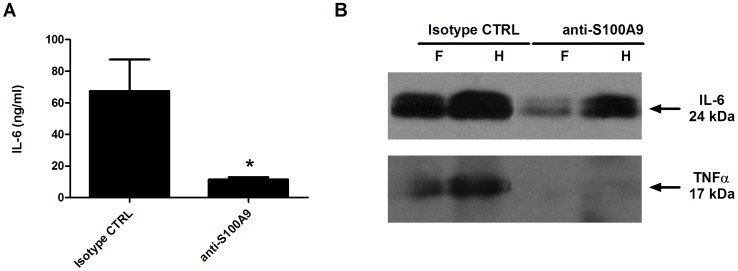
S100A9 treatment decreases the secretion of pro-inflammatory cytokines (A) 5 days after LPS injection, sera were collected and tested for IL-6 by ELISA. Values are the mean ± SEM of 10 mice. *p<0.05, t test (n = 10 serum samples/group) (B) Thirty micoliters from a pool of 5 paw homogenates were separated by sodium dodecyl sulfate polyacrylamide gel electrophoresis and tested for the presence of IL-6 and TNF-α. F: forepaw, H: hind paw.

RA is characterized by the presence of excessive concentrations of several pro-inflammatory cytokines like TNFα and IL-6. We therefore investigated the effect of anti-S100A9 on cytokine levels. Serum was collected 4 h after LPS injection and at the peak of inflammation (day 5), and IL-6 and TNFα concentrations were measured using ELISA (Fig. 5A). No cytokines were detected in sera 4 h after LPS injection.(data not shown). Interestingly, the serum concentration of IL-6 at day 5 was approximately 6 times lower in mice treated with anti-S100A9 (11.3 ng/ml compared to 67.4 ng/ml, respectively; Fig. 5A). TNFα was not detected at day 5 in mice sera.

In contrast, both IL-6 and TNFα were detected by western blot in frozen paw homogenates on day 5 after LPS injection (Fig. 5B). IL-6 expression was reduced and TNFα was almost completely absent in paws of mice treated with anti-S100A9. Thus, treatment with anti-S100A9 resulted in a sharp reduction of cytokines in serum and at the site of inflammation.

### S100A9 Increases Neutrophil Transmigration by Promoting Cell Adhesion

**Figure 6:**
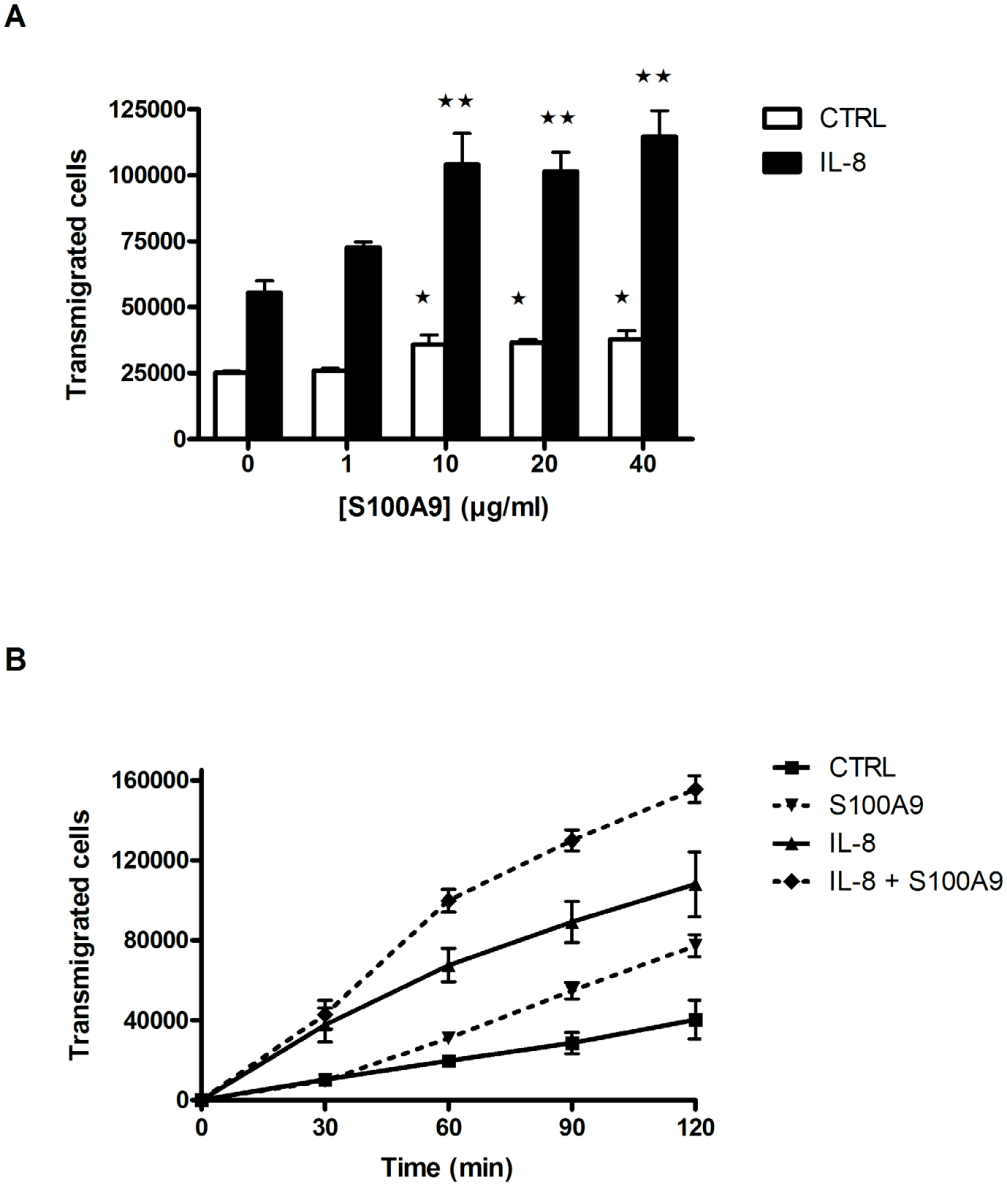
Neutrophil migration across HUVECs in response to S100A9. A) Increasing concentrations of S100A9 were added in the upper wells with neutrophils. IL-8 (5 ng/ml) or buffer was added to the lower wells and neutrophils were allowed to migrate for 2 h at 37°C. Data are the mean ± SEM of 3 experiments using neutrophils from different donors. *p<0.05, **p<0.01, one-way ANOVA, Dunnett’s multiple comparison test. B) S100A9 prolongs the time for neutrophil migration across endothelial cells. S100A9 (40 µg/ml) was added to the upper well with neutrophils. IL-8 (5 ng/ml) or buffer was added to the lower well and every 30 min for up to 2 h the upper wells were moved to new lower wells. The number of transmigrated cells was determined as described in Materials and Methods. Data shown represent the mean ± SEM of at least 3 experiments using neutrophils from different donors.

The effect of S100A9 on neutrophil migration was investigated to understand the mechanism of action of anti-S100A9. In addition to its presence at the site of inflammation, S100A9 is also found in blood where it has been hypothesized to actively participate in leukocyte migration [24]. To mimic the presence of S100A9 in the blood, different concentrations of S100A9 were incubated with neutrophils on the luminal side of endothelial cells grown on Transwell filters, while the chemoattractant IL-8 (5 ng/ml) was added on the basolateral side side to attract the cells. Cell migration was allowed to proceed for 2 h at 37°C. S100A9 significantly increased neutrophil migration, independent of the presence of IL-8 (Fig. 6A). The lowest S100A9 concentration at which this effect was observed was 1 µg/ml, and the maximal effect was observed at 10 µg/ml. These concentrations are in the range observed in sera of patients with inflammatory diseases. These data indicate that the presence of S100A9 in the serum is sufficient to weakly stimulate transendothelial migration even in absence of pro-inflammatory molecules known to attract and promote the extravasation of neutrophils. However, the response to S100A9 is additive in presence of chemotactic agents, and more neutrophils can cross the barrier when they are exposed to these DAMPS.

**Figure 7:**
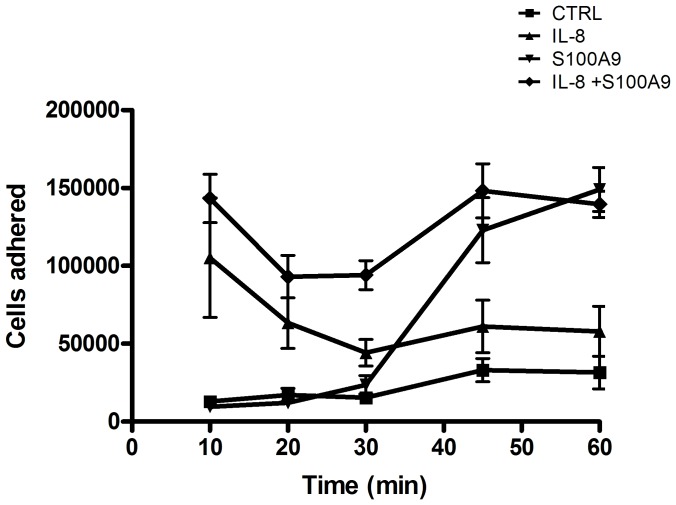
S100A9 increases neutrophil adhesion to fibrinogen. Neutrophils were incubated with S100A9 (40 µg/ml) or IL-8 (5 ng/ml) alone or in combination and allowed to adhere to fibrinogen for different incubation periods at 37°C. The number of adhered neutrophils was determined as described in Materials and Methods. Data shown represent the mean ± SEM of at least 3 experiments using neutrophils from different donors.

**Figure 8:**
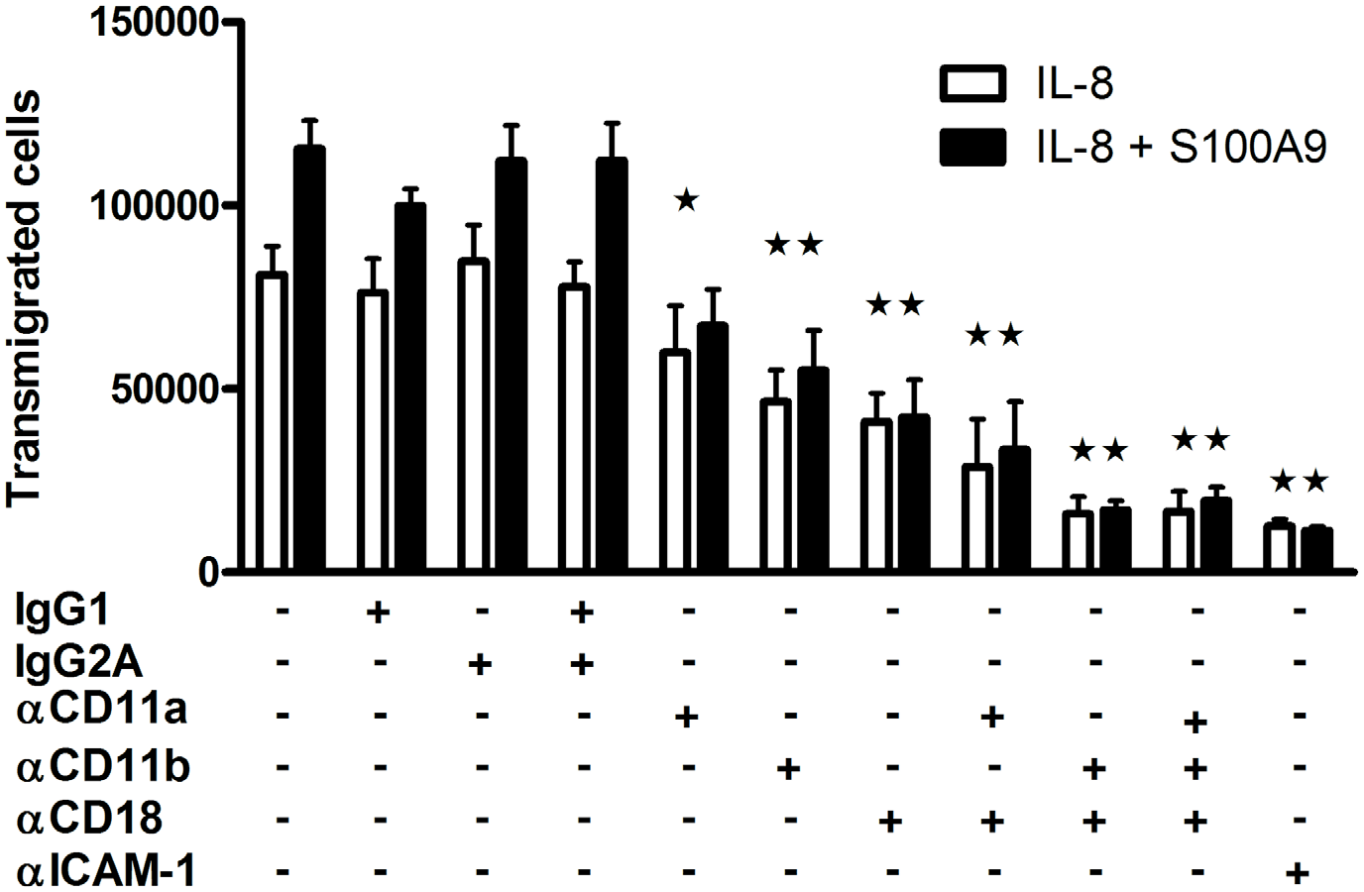
The effect of S100A9 on neutrophil migration is abolished by anti-CD11b and anti-CD18 antibodies. S100A9 (40 µg/ml) +/− antibodies against CD11a, CD11b, and CD18, or the isotype control were added to the upper wells with neutrophils. IL-8 (5 ng/ml) or buffer was added to the lower wells and neutrophils were allowed to migrate for 2 h at 37°C. The number of transmigrated cells was determined as described in Materials and Methods. Data shown represent the mean ± SEM of at least 3 experiments using neutrophils from different donors. *p<0.05, **p<0.01, one-way ANOVA, Dunnett’s multiple comparison test.

We next investigated the individual effects of S100A9 on endothelial cells and neutrophils to decipher its mechanism of action. S100A9 failed to stimulate the expression of adhesion molecules on endothelial cells or to increase endothelial cell permeability (data not shown). However, S100A9 stimulated prolonged adhesion of neutrophils to the β2 integrin substrate fibrinogen. While IL-8 induced a rapid and transient adhesion to fibrinogen, S100A9 stimulated a delayed, but prolonged adhesion of neutrophils (Fig. 7). Interestingly, this effect of S100A9 was first detected after 30 minutes of stimulation; these kinetics are similar to those of the enhancement of neutrophil transmigration in the presence of S100A9 (Fig. 6B). The role of β2 integrins in the promotion of neutrophil transendothelial migration by S100A9 was confirmed by blocking neutrophil transmigration using various β2 integrin-specific Abs (Fig. 8). Thus, the reduction of granulocyte migration in anti-S100A9-treated animals can be at least partially explained by the inhibition of serum S100A9-promoted leukocyte transmigration.

### S100A9 Induces Cytokine Secretion by Human Monocytes

**Figure 9:**
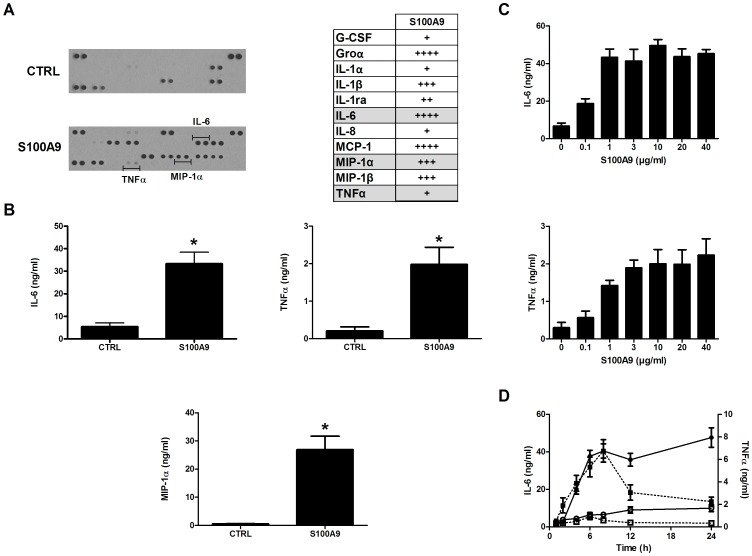
S100A9 induces the secretion of cytokines by monocytes. Cells (1 × 10^6^ cells/ml) were incubated with S100A9 (10 µg/ml) for 24 h. Supernatants were harvested and cytokines were measured by (A) cytokine arrays or (B) ELISA. CTRL: unstimulated cells. Values are the mean ± SEM of 5 different experiments. *p<0.05, t test. (C) Monocytes were stimulated with different concentrations of S100A9 (0.1–40 µg/ml) and IL-6 or TNF-α concentrations were measured in the supernatant. (D) Monocytes were incubated with 10 µg/ml of S100A9 and IL-6 or TNF-α concentrations were assessed as a function of time. Solid line: IL-6 dosage (open circles: unstimulated cells, solid circles: S100A9 stimulated cells), dotted line: TNF-α (white squares: unstimulated cells, solid squares: S100A9 stimulated cells).

To examine whether the decrease in cytokine levels could be due to a direct effect of S100A9 on cytokine secretion, human monocytes were incubated with 10 µg/ml of recombinant human S100A9 for 24 h, and supernatants were analyzed using cytokine arrays. S100A9 was found to induce the secretion of several pro-inflammatory cytokines including IL-1β, IL-6, and TNF-α, as well as chemokines such as growth-related oncogene α, IL-8, monocyte chemotactic protein-1, and MIP-1α (Fig. 9A). These results were confirmed using ELISA to detect IL-6, TNFα, and MIP-1α (Fig. 9B). Secretion of both IL-6 and TNFα was dose-dependent, with maximal secretion detected at 1 µg/ml of S100A9, a concentration found in the synovial fluid of arthritis patients (Fig. 9C). Secretion of both cytokines was maximal after 6 to 8 h of stimulation, after which time the concentration of TNFα diminished while the concentration of IL-6 remained constant (Fig 9D). Interestingly, S100A9 failed to stimulate the secretion of cytokines from neutrophils (data not shown).

## Discussion

The key role played by DAMPs in acute and chronic inflammation is well known. The DAMPs S100A9 and S100A8/A9 actively participate in acute inflammatory processes [14], [19], [20], [25], [26]. They are highly upregulated in the serum and inflamed tissues of patients with chronic inflammatory diseases like RA [10], [27], [28]. Indeed, it has been previously demonstrated that monocytes and neutrophils infiltrating the synovium express S100A9 and calprotectin [29], and both proteins are found at high concentrations in the cartilage-pannus junction, which is the main site of cartilage destruction and bone erosion [23], [27]. Moreover, Shunahori et al have shown that S100A8/A9 secreted from tissues macrophage may amplify proinflammatory cytokine responses through the activation of NF-κB and p38 MAPK pathways [30]. These clinical observations and previous studies suggest that S100A9 and calprotectin participate in innate immunity-related mechanisms observed in RA partly through their local production at inflamed joints. Decreased arthritis score was recently reported in S100A9-null mice compared to wild-type animals in antigen-induced arthritis [31], while no significant impact of S100A9 gene deletion has been reported in the K/BxN arthritis model [32]. These discrepancies might be explained by the effect of S100A9 gene deletion on S100A8. Indeed, S100A8 exhibit anti-inflammatory functions once oxidized [33], and this activity is likely not present in S100A9-deficient mice as these mice do not express S100A8 protein. In addition, both S100A8 and S100A9 have intracellular and extracellular activities which are equally affected by gene deletion. We therefore elected to specifically deplete S100A9 using an antibody to address the extracellular role of S100A9 in arthritis.

In the present study, we have identified S100A9 as a key player in the initiation and progression of arthritis. Direct inhibition of S100A9 decreased swelling and protected bone/cartilage integrity in a murine LPS-CIA model. S100A9 appears to contribute to inflammation and joint destruction not only by promoting neutrophil transendothelial migration but also by inducing the secretion of pro-inflammatory cytokines (as described in the studies of Sunahori et al [30]) and matrix-modifying enzymes by innate immune cells.

In RA, inflammatory mediators derived from resident and innate immune cells play crucial roles in amplifying and perpetuating inflammation, independent of the trigger. Downregulating this circuit may prevent acute phase inflammation and limit the progression of the disease. Here, we have shown that S100A9 and calprotectin are detected early in serum after the induction of arthritis. Interestingly, while anti-S100A9 eliminated the presence of these molecules in the serum, anti-TNFα did not prevent their release, suggesting that secretion of TNFα occurs after the export of these proteins. Neutrophils are rapidly mobilised to the inflammatory site, and since they are rich in S100A9, they can swiftly release S100A9 and calprotectin in the absence of gene transcription [34], [35]. Once secreted, S100A9 and calprotectin can stimulate neighbour innate immune or non-immune cells to produce inflammatory mediators like TNFα, IL-6, and IL-1β, which are known to exacerbate and sustain inflammation. Taken together, our results confirm that S100A9 stimulates the production of pro-inflammatory cytokines and chemokines such as TNFα, IL-6, and MIP-1α by monocytes. These mediators participate in the induction of the acute arthritis phase as well as in disease progression, resulting in infiltration of immune cells into the joints, localized secretion of cytokines and tissue-degrading enzymes, and ultimately cartilage and bone destruction.

Treatment with anti-S100A9 was associated with a decrease in cartilage and bone erosion, both of which are closely related to immune cell infiltration. Indeed, treatment with anti-S100A9 strongly decreased the infiltration of neutrophils and monocytes into the paws of arthritic mice. These results highlight the participation of S100A9 and calprotectin in leukocyte migration toward inflammatory sites. Indeed, both are deposited on endothelium by neutrophils at inflammatory sites [36], [37] and are presumed to play a role in neutrophil adhesion to endothelium. Here we further demonstrate that S100A9 increased neutrophil transmigration, probably by prolonging the time window for the movement of neutrophils across the endothelial barrier through the extended activation of β2 integrins. The delayed migration effect of S100A9 suggests that it may induce inside-out signaling, which is required to increase activation of β2 integrins [38], or promote the release of secondary activators such as lipid mediators, which are known to upregulate the ligand-binding activity of β2 integrins [39], [40]. These results are consistent with those from Eue et al., which demonstrated that the addition of anti-S100A9 or anti-S100A8/A9 reduced monocyte migration across endothelial cells [17]. Based on these observations, it is clear that S100A9 is linked to the migration of leukocytes, particularly neutrophils, to the site of inflammation.

Recruitment of innate immune cells into joint articulations leads to local inflammation through the release of proinflammatory mediators that activate resident immune and non-immune cells in the synovium. Cytokines like TNFα and IL-6 are overproduced in RA synovial tissues and play multiple roles in the release of other inflammatory mediators, chemoattractants, and matrix-modifying enzymes. As expected, high levels of TNFα and IL-6 were detected in the paws of arthritic mice. Interestingly, when mice were injected with anti-S100A9, we observed a reduction in TNFα that can be correlated with the decrease in monocyte/granulocyte infiltration. Similar effects were obtained for IL-6, with the difference that low-level expression of IL-6 was still detected in the paws of animals treated with anti-S100A9 Abs. This observation could be partly explained by the fact that synovial fibroblasts produce considerable amounts of this cytokine in the absence of stimuli, and this participates in steady-state bone homeostasis [41], [42].

Cartilage degradation and bone erosion are due to a complex interplay between immune cell infiltration, cytokines, matrix-modifying enzymes such as matrix metalloproteinases (MMPs), and osteoclast/osteoblast turnover. It has been recently demonstrated that S100A8 and S100A9 play active roles in these three aspects. Indeed, S100A9−/− mice, which also lack the S100A8 protein, show reduced bone erosion in antigen-induced arthritis through a mechanism partly related to a decrease in osteoclasts [31], [43]. Moreover, S100A8- and S100A9-mediated stimulation of human chondrocytes induces the expression of pro-inflammatory cytokines and MMPs that promote cartilage degradation and prevents the formation of new cartilage matrix molecules [44]. In addition, it was recently reported that TNFα- and IL-17-mediated joint erosion depends on the expression of S100A8, MMPs, and a disintegrin and metalloproteinase with thrombospondin motifs [45]. These findings reinforce our observations that the neutralization of S100A9 and calprotectin preserves joint integrity in arthritis. Indeed, we observed little or no bone erosion and cartilage destruction in groups treated with anti-S100A9 Abs. These observations are consistent with decreased infiltration of neutrophils and monocytes, which are known to secrete matrix-modifying enzymes involved in joint degradation. S100A9 may have a direct role in the release of MMPs. Indeed, we previously showed that stimulation of neutrophils with S100A9 induces the degranulation of secretory and specific/gelatinase granules containing MMP9 or collagenase [46].

Based on present and previous studies, we propose a model linking S100A9 and calprotectin in the development of acute RA and the exacerbation of inflammation in the synovium. Briefly, innate immune cells, such as monocytes and neutrophils, are first activated by inflammatory stimuli, triggering the release of S100A9 and calprotectin. These DAMPs can act in either an autocrine or paracrine manner to favor cell migration towards the site of inflammation. Once in the synovium, infiltrated cells release pro-inflammatory mediators that subsequently activate resident immune and non-immune cells, resulting in a feed-forward loop of inflammation that ultimately causes tissue destruction. Numerous DAMPs including S100A9 and calprotectin are among the pro-inflammatory factors liberated by activated resident cells. Indeed, the genes encoding S100A8 and S100A9 are induced in various cell types in response to inflammatory agonists like epithelial and endothelial cells, keratinocytes, fibroblasts and macrophages [47], [48]. The local presence of S100A9 and calprotectin in the synovium can further exacerbate the destructive inflammatory environment in the joint by activating the proliferation of fibroblast-like synoviocytes, as has already been reported for fibroblasts [49]. Fibroblast-like synoviocytes are one of the main classes of effector cells in RA. Their expansion and infiltration into cartilage directly contributes to tissue destruction via the secretion of matrix-modifying enzymes and pro-inflammatory mediators [50]. Through activation of osteoclasts, S100A8 and S100A9 may also participate in bone remodeling and erosion observed in RA. Therefore, we believe that S100A9 and calprotectin participate in an amplification loop driving inflammation-mediated tissue destruction in the joint. Neutralizing the activity of these molecules might be helpful in the treatment of inflammation associated with RA, as well as with other chronic inflammatory and autoimmune diseases where S100A9 and calprotectin are oversecreted. This conclusion is strongly supported by a recent publication showing that the absence of S100A8 and S100A9 precludes the development of autoreactive CD8^+^ T cells in a lupus erythematous model [51].

In conclusion, we identified S100A9 and calprotectin as important players in the pathogenesis of RA. Our study provides additional evidence suggesting that these proteins represent an important target in the treatment of rheumatoid arthritis.
